# A New Combination Formula for Treatment of Fungal Keratitis: An Experimental Study

**DOI:** 10.1155/2014/173298

**Published:** 2014-04-29

**Authors:** Hala Mohamed El-Mofty, Mohamad Amr Salah Eddin Abdelhakim, Mohamed Farid El-Miligi, Mohamed A. El-Nabarawi, Islam Ahmed Hamed Khalil

**Affiliations:** ^1^Department of Ophthalmology, Kasr Al Ainy Hospital, Cairo University, Cairo, Egypt; ^2^Department of Pharmaceutics and Industrial Pharmacy, Faculty of Pharmacy, Cairo University, Cairo, Egypt; ^3^Department of Pharmaceutics and Industrial Pharmacy, College of Pharmacy, Misr University for Science & Technology, 6 October City, Giza, Egypt

## Abstract

*Objective*. To formulate and evaluate slow release ketoconazole and ketorolac to treat fungal keratitis and associated inflammation. *Methods*. Experimental study with the following outcome measures. *Pharmaceutical Evaluation*. Mucoadhesive gels containing ketoconazole and ketorolac were used. *Microbiological in vitro evaluation* was performed using cup method. *In vivo evaluation* was performed on 24 rabbits divided into 2 groups, 12 rabbits each, group A (fast release formula; 6 times daily) and group B (slow release formula; 3 times daily). Each group was divided into two subgroups (6 rabbits each). Both eyes of rabbits were inoculated with *Candida albicans*. The left eye of all rabbits received the combination formulae. The right eye for one subgroup received ketoconazole as control 1 while the other subgroup received placebo as control 2. Clinical follow-up was done and, finally, the corneas were used for microbiological and pathological evaluation. *Results*. Gels containing high polymer concentration showed both high viscosity and mucoadhesion properties with slower drug release. The infected eyes treated with slow release formula containing both drugs showed better curing of the cornea and pathologically less inflammation than eyes treated with fast release formula. *Conclusion*. Slow release formula containing ketoconazole and ketorolac showed higher activity than fast release formula against fungal keratitis and associated inflammation.

## 1. Introduction

Limited absorption of drug through lipophilic corneal barrier is mainly due to short precorneal residence time related to tear turnover, rapid nasolacrimal drainage of instilled drugs from the tear turn fluid, and nonproductive absorption of drug through the conjunctiva. To overcome this problem, designing a drug delivery system with ability to retain in the eye for long period of time will increase the absorbed proportion of the drug [1].

Ketoconazole is an imidazole antifungal agent with some antibacterial activity. As with other imidazoles, it has five-membered ring structure containing two nitrogen atoms. It inhibits the synthesis of ergosterol by blocking the action of 14-alpha-demethylase. Although Natamycin eye drop is the only product in the international market, it is only useful for superficial infections, penetrates the cornea poorly, and is much less effective against* Candida* species or deep fungal corneal infections [2].

In a study by Hemady and colleagues on rabbits, ketoconazole levels in the cornea and aqueous humor were high after topical or subconjunctival administration and increased markedly (especially in the cornea) if the corneal epithelium had been debrided before administration of the drug [3].

Ketorolac tromethamine is nonsteroidal anti-inflammatory drug. It owns its principle effect to inhibit cyclooxygenase enzyme which transforms arachidonic acid into prostaglandins. Using ketorolac, as topical anti-inflammatory in eye inflammation, was found to be effective and safer alternative than topical steroids. The addition of anti-inflammatory drug to antifungal drug is very important to treat inflammation, ulcer, and edema which are associated with fungal eye infection [4].

So, the aim of this work was to formulate slow release ketoconazole using mucoadhesive polymers in order to achieve higher absorption and to prolong the duration of its therapeutic effect. We also aimed to formulate a combination of ketoconazole and ketorolac tromethamine as anti-inflammatory drug to treat the inflammation associated with fungal keratitis.

## 2. Materials and Methods

This is an experimental study conducted at Kasr Al Ainy Faculty of Medicine, Cairo University. It was divided into 3 sections.

The first section was concerned with the pharmaceutical evaluation of the formulations. The second section was concerned with the* in vitro* microbiological study, while the third section was concerned with* in vivo* microbiological study.

### 2.1. Pharmaceutical Evaluation

Mucoadhesive gels containing ketoconazole (Oman Chemicals & Pharmaceuticals LLC, Al Buraimi, Sultanate of Oman) and ketorolac (Hetero Drugs Limited, Erragadda, Hyderabad-AP, India) in Carbopol 940 (B.F., Goodrich Chemical Company, OH, USA) or methylcellulose (Dow Chemical Company, USA) or hydroxypropyl methylcellulose (Aqualon, UK) were prepared in different concentrations. The prepared formulations were evaluated in terms of rheology, pH measurement, mucoadhesion, effect of sterilization,* in vitro* drug release in Sorensen phosphate buffer pH (7.4), and stability study. After evaluation of the efficacy and potency, laboratory study was done.

### 2.2. *In Vitro* Antimicrobial Study

*Preparation of the Studied Strain.* The fungal strain was identified and prepared in sterile saline as follows:* Candida* species were obtained from the Sabouraud dextrose agar to prepare cell suspension in sterile normal saline which was adjusted to McFarland number 1.0 turbidity, and the suspensions were inoculated to give a final concentration of 5 × 10^5^ cells/mL. This suspension was used for both* in vitro* susceptibility and corneal animal inoculation.

*(1) Activity Study of Antifungal Evaluation (Cup Method). *The selected formulae were tested in triplicate manner using agar cup diffusion method against* Candida albicans* strain. Cups of 10 mm diameter were made aseptically in Sabouraud dextrose agar after being inoculated with tested fungal suspension strain by spreading on the agar surface. From each formula of the antifungal drug and blank (formula without antifungal drug), 100 mg was filled into the cups with sterile syringe. The inhibition zone for each cup was measured and compared to the blank formulae [5, 6]. 

*(2) Drug Release Study from the Antifungal Formulae Using the Cup Method. *The selected formulae were tested in triplicate manner using agar cup diffusion method against* Candida albicans* strain. Cups of 10 mm in diameter were made aseptically in Sabouraud dextrose agar after being inoculated with tested fungal suspension strain by spreading on the agar surface. From each drug formula, 50, 100, and 150 mg were filled to the cups with a sterile syringe.

The diameter of inhibition zone of each cup was measured and plotted against the formula weight. The Slope, which represents the release rate constant, and the intercept were calculated. 

*(3) Comparison of Release Data with Commercially Available Product. *Nizoral cream was used as a market product, which contains ketoconazole 2%. The results were compared with the practical formulae.

### 2.3. *In Vivo* Antimicrobial Study

For this study, the selected formulae were formula PF44 (CL 940 1%) as a rapid release formula and formula PF51 (HPMC 4%) as a slow release formula. PF 44 is a polymer formula consisting of 2% ketoconazole solid dispersion, 0.5% ketorolac, and 1% Carbopol 940. PF 51 is a polymer formula consisting of 2% ketoconazole solid dispersion, 0.5% ketorolac, and 4% hydroxyl-propyl-methylcellulose.

The study was performed on 24 rabbits, from the animal house of Kasr Al Ainy Cairo University, weighing 1.5–2.5 kg with no previous eye lesions, receiving green food only, according to the ARVO Statement for the Use of Animals in Ophthalmic and Vision Research.

The 24 rabbits were divided into 2 groups with group A assigned for PF 44 while group B was assigned for PF 51. Each group consisted of 12 rabbits and was divided into two subgroups; each of which contained 6 rabbits. Both eyes of each rabbit were inoculated with fungi (*Candida albicans*). In each group, the left eye of all rabbits received the combination formulae. The right eye for one subgroup received the ketoconazole formulae as a control 1 while the other subgroup received placebo as a control 2.

The rabbits were sedated by ketamine. The rabbits' corneas were marked with trephine 7 mm, and removal of surface epithelium in the form of scraping was done. Washing the eyes with topical tobramycin was made. 100 *μ*L (500,000 spores) of the spore suspension of fungal strain was injected into the corneal stroma.

The rabbits' eyes were examined every day. In addition, the eyes were photographed. The rapid release formula (PF44) was applied 6 times per day (every 4 hours), while the slow release formula (PF51) was applied 3 times per day (every 8 hours) for ten days. These formulae were started 48 hours following inoculation.

Follow-up of size and depth of corneal ulcer, and signs of inflammation in the form of severe iritis or hypopyon was done for each rabbit and recorded according to their enumeration. The animals were scarified after 12 days from inoculation.

The corneas were excised at limbal margin and each cornea was used for microbiological and pathological evaluation. The paraffin embedded sections were stained with hematoxylin eosin, periodic acid Schiff's, and special stains like Gomori's methenamine silver stain and Grams stains. The sections were studied for histologic changes, namely, ulceration, inflammation, vascular channels, necrosis, keratocyte loss, and other details. Inflammation was graded in a semiquantitative way as mild, moderate, or severe based on the density of the inflammatory cells. We looked for the absence or the reduced number of the keratocytic nuclei in the corneal stroma surrounding the zone of inflammation. 

*Statistical Analysis.* Data were statistically described in terms of mean ± standard deviation (±SD) or frequencies (number of cases) and percentages when appropriate. Chi square (Fisher's exact) test was used to evaluate associations between qualitative data. All *P* values less than 0.05 were considered statistically significant. All statistical calculations were done using computer programs SPSS (Statistical Package for the Social Science; SPSS Inc., Chicago, IL, USA) version 18 for Microsoft Windows.

## 3. Results

### 3.1. Pharmaceutical Evaluation

All gel formulations had pH 7. Gels containing high polymer concentration showed both high viscosity and mucoadhesion properties with slower drug release, which all make them good candidates for further* in vivo* investigation. Formula 1 had rapid release pattern which offered 6 times daily dose frequency while formula 2 had slow release pattern which offered 3 times daily dose frequency. Evaluation of the sterile formulae revealed that both formulae were very stable after sterilization according to all parameters. Also both formulae showed a good physical stability after applying stability study.

### 3.2. *In Vitro* Antimicrobial Study****

**Figure 1:**
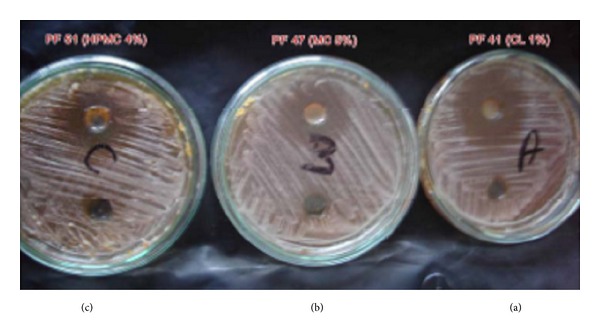
Picture of antimicrobial activity study for different formulae against* Candida albicans* using Cup Method: (a) polymer formula (PF) 44, (b) PF 47, and (c) PF 51.

*(1) Activity Study of Antifungal Evaluation (Cup Method).* Table 1 and Figure 1 show the results of formulae activity against* Candida albicans* strains. 

**Figure 2:**
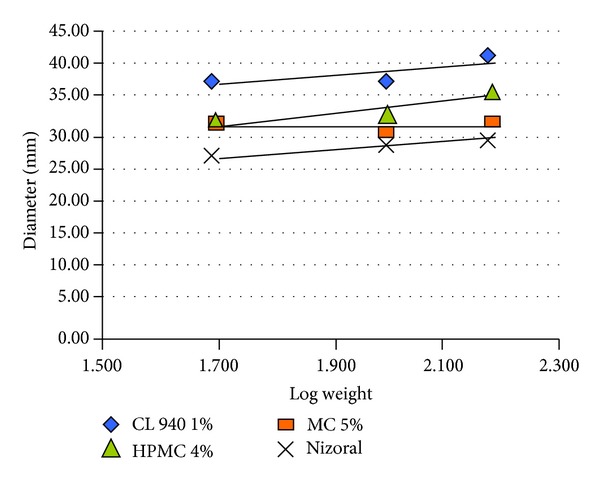
Drug release study from different formulae and Nizoral cream against* Candida albicans* using Cup Method. CL**:** Carbopol, MC**:** methylcellulose, and HPMC**:** hydroxy-propyl-methylcellulose.

**Figure 3:**
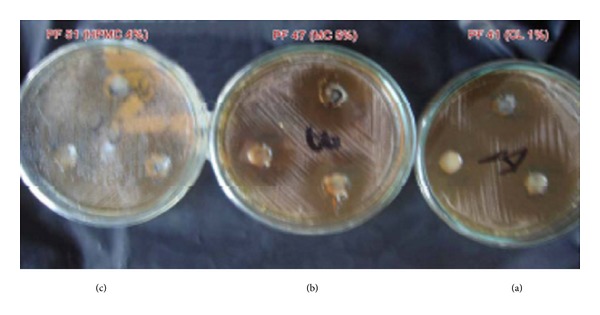
Picture of drug release study of different formulae against* Candida albicans* using Cup Method: (a) polymer formula (PF) 44, (b) PF 47, and (c) PF 51.

*(2) Drug Release Study from Antifungal Formulae Using the Cup Method.* Table 2 and Figures 2 and 3 showed the results of formulae release rate against* Candida albicans* using the Cup Method. 

*(3) Comparison of Release Data with Commercially Available Product.* Table 2 and Figure 3 show the results of different formulae and of Nizoral cream release rates against* Candida albicans* using the Cup Method.

So, PF 44 had a high inhibition zone and release rate in comparison with both other formulae and Nizoral cream. In addition, both PF 47 and PF 51 had similar efficiency but PF 51 had a higher release rate.

### 3.3. *In Vivo* Antimicrobial Study****

**Figure 4:**
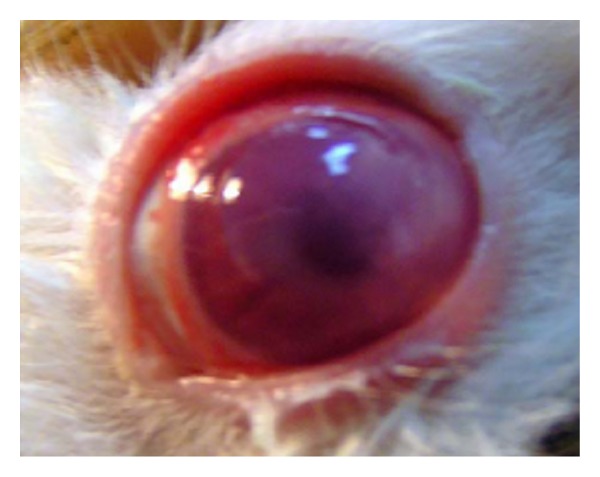
Rabbit's eye after 48 hours of inoculation that shows the cornea with deep stromal infiltrate and surrounding stromal oedema and inflammation.

*(1) Clinical Profile. *All rabbits after 48 hours from inoculation started receiving treatment. The rabbits' corneas before treatment suffered from deep stromal infiltration and a surrounding stromal edema and inflammation (Figure 4).  Table 3 shows the percentage of cured animal treated with both formulae (*P* < 0.001). The cure was judged by improvement in size and depth of ulcer and hypopyon with improvement of stromal edema and obvious healing epithelium.

*(2) Microbiology. *The rabbits' corneas from both groups were microbiologically tested and the results are shown in Table 4. 

**Figure 5:**
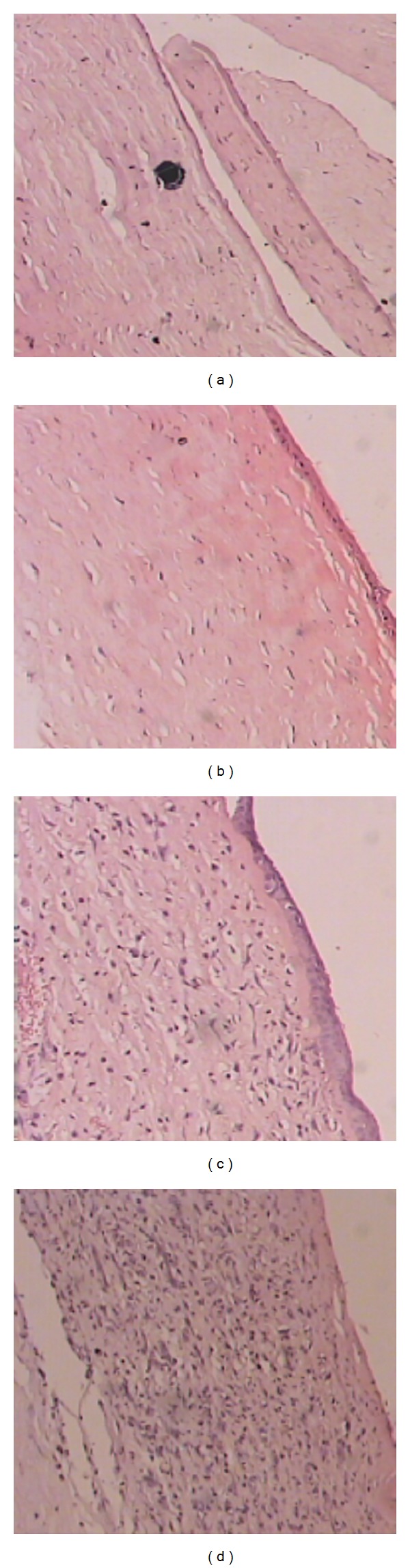
Different corneal sections showing different inflammatory degrees (paraffin embedded sections stained with hematoxylin eosin). (a) Corneal section showing* Candida albicans* spore in the deep stroma. (b) Corneal section showing polymorph nuclear leucocytes (PMN) (+). (c) Corneal section showing PMN (++). (d) Corneal section showing PMN (+++).

*(3) Histopathology. *The histological changes observed in the sections of the excised corneal tissues are shown in Figure 5 and Table 5. The degree of inflammation was classified according to the amount of polymorph nuclear leucocytes (PMNs) as follows: PMN (+) = mild inflammation, PMN (++) = moderate inflammation, and PMN (+++) = severe inflammation.

## 4. Discussion

Infections of the cornea are potentially blinding diseases. Despite best efforts with early diagnosis and specific antimicrobial treatment, about one-third of cases require surgical intervention [7]. Those which respond to medical treatment result in scarring which may lead to varying degree of visual disability. The tissue destruction in infectious keratitis, irrespective of the etiologic agent, is the compound effect of cytokines and inflammatory mediators released by the microorganism, host inflammatory cells, and the metalloproteases that act on the collagen [8]. The aim of treating corneal infections is not only to control the infection but also to restrict the tissue damage so that the corneal stroma maintains the transparency, thus retaining its visual function. The requirement of a topical application of antifungal drug and anti-inflammatory drug combinations for treating fungal keratitis has forced itself by the increased incidence of fungal keratitis, which led to blindness if not treated properly.

A study by El-Nabarawi et al. on ketoconazole and fluconazole as topical dosage form showed promising results. After a clinical study using both drugs, ketoconazole had a better result than fluconazole. However, it was observed that the patient suffered from eye redness [9].

Therefore, in this study, we decided to include ketorolac tromethamine as an anti-inflammatory drug during treatment with ketoconazole to relieve the inflammation. In our study, the left eye of the 24 rabbits, which received the combination formula, showed less inflammatory cells than the right eye of the first subgroup, which received ketoconazole alone, which consequently showed less inflammatory cells than the placebo group.

Though wound healing following surgical procedures including routine keratoplasty and refractive surgeries is well documented, [10] healing after an infectious etiology is not well documented. It was observed the loss of keratocytes in deeper stroma in* Acanthamoeba *keratitis and had proposed that one of the mechanisms for this loss was apoptosis of keratocytes. It was observed since then that the paucity of stromal keratocytes in the zone surrounding the infected area in all types of corneal infections prompts us to speculate that it is possibly a host mediated response to an infectious process [11].

Keratitis is usually treated with a topical or intrastromal antifungal medication, sometimes in conjunction with subconjunctival injections of the same drug and/or oral antifungal [12]. Natamycin applied on an hourly basis is the preferred polyene for topical administration as the deoxycholate salt in amphotericin B is toxic to the cornea [13]. Collagen shields soaked in amphotericin B have been used with success with* Aspergillus* keratitis [14]. The topical use of the antiseptic agent chlorhexidine gluconate at a 0.2% concentration showed efficacy* in vitro* [15] and in patients with fungal keratitis [16].

In our study, the highest cure rate was obvious in the eyes which received the slow release ketoconazole (100%), followed by the eyes which received the slow release combination formula (83.3%), and then the eyes which received the rapid release ketoconazole or its combination formula (66.7% each), with a significant difference between these groups (*P* < 0.001).

Microbiological examination of the excised corneas showed no organism growth in the eyes treated with rapid release formula and slow release formula and in those treated with slow release ketoconazole. These results were similar to the clinical profile, which revealed that slow release formula scored better results either by the combination or by ketoconazole alone.

## 5. Conclusion

From all of these results, the slow release formula may be concluded to have a better cure percent in clinical profile and negative growth of* Candida albicans* in microbiological test. In addition, it showed an absence of mild inflammation on histopathology. This makes this formula a good candidate for further clinical trials on human corneas.

Future studies using this slow release formula to treat human fungal keratitis are recommended; however, caution is a must as ketoconazole toxicity on human corneas has been reported.

In this work, studying the toxicity of ketoconazole was not our target. However, Foster and his colleagues (1981) studied the ocular toxicity of topical 1% solutions or suspensions of amphotericin B, flucytosine, miconazole nitrate, and ketoconazole on rabbits' eyes. Flucytosine, miconazole, and ketoconazole did not retard the closure of 8.5 mm corneal epithelial defects; amphotericin B greatly retarded the closure of such defects. Amphotericin B produced dramatic pathologic changes in this model; these changes worsened with each day of therapy. Ketoconazole produced modest biomicroscopically and histologically detectable pathologic changes in the regenerating corneal epithelium; flucytosine and miconazole did not produce such changes [17].

## Figures and Tables

**Table 1:** Activity study of different formulae against *Candida albicans* using Cup Method.

Formula	Content	Mean diameter (mm)
PF 44	CL 940 1%	37 ± 1.2
PF 47	MC 5%	29 ± 0.9
PF 51	HPMC 4%	32 ± 1.1

PF: polymer formula; CL: Carbopol, HPMC: hydroxy-propyl-methyl-cellulose; MC: methylcellulose.

**Table 2:** Drug release study from different formulae and Nizoral cream against *Candida albicans* using Cup Method.

Formula weight (mg)	Mean diameter (mm)			
	PF 44	PF 47	PF 51	Nizoral
50	37.33 ± 2.08	31 ± 1.15	31.33 ± 1.15	27 ± 0.0
100	38.33 ± 1.15	31.33 ± 1.73	33 ± 1.0	29 ± 0.0
150	41.33 ± 1.15	32 ± 2.0	35.33 ± 1.53	29.33 ± 0.58
Slope	7.839	1.988	8.077	5.079
Rate constant	3.404	0.863	3.507	2.205
Intercept	23.65	27.55	17.40	18.50
Molecular weight (mg)	0.018	1.49*E* − 09	0.12	0.021

PF**:** polymer formula.

**Table 3:** Effect of different antifungal formulae on rabbits' corneas.

	Left eye (*n* = 12)				Right eye (*n* = 12)							
Antifungal formula					Control 1 (*n* = 6)				Control 2 (*n* = 6)			
	Cured		Not cured		Cured		Not cured		Cured		Not cured	
	*N*	%	*n*	%	*N*	%	*n*	%	*n*	%	*n*	%
Group A (rapid release PF 44)	8	66.7	4	33.3	4	66.7	2	33.3	0	0	6	100

Group B (slow release PF 51)	10	83.3	2	16.7	6	100	0	0	0	0	6	100

*P* value	**<0.001**											

*P* value < 0.05 is considered statistically significant.

PF: polymer formula.

**Table 4:** Susceptibility test of rabbits' corneas showing fungal growth.

Antifungal formulae	Left eye	Right eye	
		Control 1	Control 2
Rapid release PF 44	No growth	Growth	Growth
Slow release PF 51	No growth	No growth	Growth

PF: polymer formula.

**Table 5:** Histopathological results of rabbits' corneas after being sacrificed.

Antifungal formulae	First subgroup		Second subgroup	
	Left eye	Right eye (ketoconazole)	Left eye	Right eye (placebo)
Rapid release PF 44	Stromal macrophages PMN (+)	Stromal macrophages PMN (++) *Candida albicans *	Stromal macrophages PMN (+)	Stromal macrophages PMN (+++) *Candida albicans *

Slow release PF 51	Stromal macrophages PMN (+)	Stromal macrophages PMN (++) *Candida albicans *	Stromal macrophages	Stromal macrophages PMN (+++) *Candida albicans *

PMN: polymorph nuclear leucocytes; PF: polymer formula.

## References

[1] Maurice DM, Mishima S, Sears ML (1984). Ocular pharmacokinetics. *Handbook of Experimental Pharmacology*.

[2] Skiba M, Skiba-Lahiani M, Marchais H, Duclos R, Arnaud P (2000). Stability assessment of ketoconazole in aqueous formulations. *International Journal of Pharmaceutics*.

[3] Hemady RK, Chu W, Foster CS (1992). Intraocular penetration of ketoconazole in rabbits. *Cornea*.

[4] Martindale (2007). *The Complete Drug Reference*.

[5] Bachhav YG, Patravale VB (2009). Microemulsion-based vaginal gel of clotrimazole: formulation, *in vitro* evaluation, and stability studies. *AAPS PharmSciTech*.

[6] Bauer AW, Kirby WM, Sherris JC, Turck M (1966). Antibiotic susceptibility testing by a standardized single disk method. *American Journal of Clinical Pathology*.

[7] Miedziak AI, Miller MR, Rapuano CJ, Laibson PR, Cohen EJ (1999). Risk factors in microbial keratitis leading to penetrating keratoplasty. *Ophthalmology*.

[8] Gopinathan U, Ramakrishna T, Willcox M (2001). Enzymatic, clinical and histologic evaluation of corneal tissues in experimental fungal keratitis in rabbits. *Experimental Eye Research*.

[9] El-Nabarawi MA, El-Mofty HM, Ahmed MH, Behiry IK (2003). Development of economic corneal formula containing azole derivative for the treatment of human fungal keratitis. *Egyptian Journal of Biomedical Sciences*.

[10] Wilson SE, Mohan RR, Hong J-W, Lee J-S, Choi R, Mohan RR (2001). The wound healing response after laser in situ keratomileusis and photorefractive keratectomy. Elusive control of biological variability and effect on custom laser vision correction. *Archives of Ophthalmology*.

[11] Vemuganti GK, Sharma S, Athmanathan S, Garg P (2000). Keratocyte loss in Acanthamoeba keratitis: phagocytosis, necrosis or apoptosis ?. *Indian Journal of Ophthalmology*.

[12] Prakash G, Sharma N, Goel M, Titiyal JS, Vajpayee RB (2008). Evaluation of intrastromal injection of voriconazole as a therapeutic adjunctive for the management of deep recalcitrant fungal keratitis. *American Journal of Ophthalmology*.

[13] Pleyer U, Grammer J, Pleyer JH (1995). Amphotericin B—level of effectiveness in the cornea. *Ophthalmologe*.

[14] Mendicute J, Ondarra A, Eder F, Ostolaza JI, Salaberria M, Lamsfus JM (1995). The use of collagen shields impregnated with amphotericin B to treat Aspergillus keratomycosis. *CLAO Journal*.

[15] Martin MJ, Rezanur Rahman M, Johnson GJ, Srinivasan M, Clayton YM (1995). Mycotic keratitis: susceptibility to antiseptic agents. *International Ophthalmology*.

[16] Rahman MR, Minassian DC, Srinivasan M, Martin MJ, Johnson GJ (1997). Trial of chlorhexidine gluconate for fungal corneal ulcers. *Ophthalmic Epidemiology*.

[17] Foster CS, Lass JH, Moran-Wallace K, Giovanoni R (1981). Ocular toxicity of topical antifungal agents. *Archives of Ophthalmology*.

